# A Real-Time Lift Detection Strategy for a Hip Exoskeleton

**DOI:** 10.3389/fnbot.2018.00017

**Published:** 2018-04-12

**Authors:** Baojun Chen, Lorenzo Grazi, Francesco Lanotte, Nicola Vitiello, Simona Crea

**Affiliations:** ^1^The BioRobotics Institute, Scuola Superiore Sant'Anna, Pontedera, Italy; ^2^Fondazione Don Carlo Gnocchi, Firenze, Italy

**Keywords:** lift detection, lift assistance, hip exoskeleton, exoskeleton control, EMG reduction

## Abstract

Repetitive lifting of heavy loads increases the risk of back pain and even lumbar vertebral injuries to workers. Active exoskeletons can help workers lift loads by providing power assistance, and therefore reduce the moment and force applied on L5/S1 joint of human body when performing lifting tasks. However, most existing active exoskeletons for lifting assistance are unable to automatically detect user's lift movement, which limits the wide application of active exoskeletons in factories. In this paper, we propose a simple but effective lift detection strategy for exoskeleton control. This strategy uses only exoskeleton integrated sensors, without any extra sensors to capture human motion intentions. This makes the lift detection system more practical for applications in manufacturing environments. Seven healthy subjects participated in this research. Three different sessions were carried out, two for training and one for testing the algorithm. In the two training sessions, subjects were asked to wear a hip exoskeleton, controlled in transparent mode, and perform repetitive lifting and a locomotion circuit; lifting was executed with different techniques. The collected data were used to train the lift detection model. In the testing session, the exoskeleton was controlled in order to deliver torque to assist the lifting action, based on the lift detection made by the trained algorithm. The across-subject average accuracy of lift detection during online test was 97.97 ± 1.39% with subject-dependent model. Offline, the algorithm was trained with data acquired from all subjects to verify its performance for subject-independent detection, and an accuracy of 97.48 ± 1.53% was achieved. In addition, timeliness of the algorithm was quantitatively evaluated and the time delay was <160 ms across different lifting speeds. Surface electromyography was also measured to assess the efficacy of the exoskeleton in assisting subjects in performing load lifting tasks. These results validate the promise of applying the proposed lift detection strategy for exoskeleton control aiming at lift assistance.

## Introduction

Repetitive lifting of heavy objects is one of the most common factors causing health problems such as low back pain (Punnett et al., 2005) and work-related musculoskeletal disorders (da Costa and Vieira, 2010). However, a lot of workers have to lift heavy loads during working. For example, the sixth European Working Conditions Survey carried out in 2015 in 35 countries revealed that 32% of workers perform tasks like carrying or moving heavy loads, and 10% of health and personal care workers (e.g., nurses) are required to lift and carry patients (Eurofound, 2016). These two proportion values change very little when compared with those in 2005 (32 and 8%) and 2010 (34 and 9%), which implies this situation has not changed for more than 10 years. In recent years, some passive exoskeletons have been developed and proved to be able to reduce the muscle activities at the low back when performing lifting tasks (Abdoli-e et al., 2006; Abdoli-Eramaki et al., 2007; Abdoli-e and Stevenson, 2008; Lotz et al., 2009; Wehner et al., 2009; Whitfield et al., 2014; Masood et al., 2016). These systems typically rely on spring-based mechanisms, designed so that the energy stored in the lowering phase is exerted back to the user in the lifting phase. Despite the positive results, such systems cannot generate high forces or torques, and are not versatile for use in tasks different from lifting; for example, when used in walking, they can hinder the movement and cause increased leg muscle activity, discomfort and muscle deconditioning (de Looze et al., 2016).

Active exoskeletons seem to be more promising in assisting workers and reducing the risk of lumbar vertebral injuries due to the higher versatility of the control system and external power source. Over the last decade, several active exoskeletons have been developed for lifting assistance worldwide (Naruse et al., 2005; Kobayashi and Nozaki, 2007; Tanaka et al., 2008; Aida et al., 2009; Kadota et al., 2009; Kobayashi et al., 2009; Muramatsu et al., 2011; Li, 2013; Yu et al., 2015) and were demonstrated to reduce the musculature effort of the back extensor muscles (Li, 2013; Muramatsu et al., 2013). However, in many cases the control systems do not independently detect the user's intention, and therefore are unable to automatically trigger the delivery of the assistance at the right moment: power assistance is usually manually triggered by users with extra joysticks or control buttons. Kobayashi and Nozaki used two buttons to control the supply and release of compressed air in the McKibben artificial muscle (Kobayashi and Nozaki, 2008). The button controller was tied to user's belt and the user could easily reach the buttons and trigger power assistance by himself. In a follow-up study, Muramatsu et al. simplified the button controller by mounting two small control switches on user's fingers. When intended to lift a load, the user was required to push the control switch to trigger power assistance (Muramatsu et al., 2013). Though this approach is simple and easy to operate, it has some limitations. First, extra devices need to be placed on user's fingers and users have to manually control the exoskeleton, which increases user's cognitive burden and introduces inconvenience to the user. Second, this approach makes lifting tasks intermittent and reduces work efficiency, which could reduce exoskeletons acceptability in work environments. Third, since user's hands are usually occupied when lifting heavy loads, mistaken operations may happen when grasping the load. For all these reasons, the development of control systems capable to automatically detect the lift movement as soon as it starts can turn out fundamental.

Compared to the studies on lifting assistance exoskeleton development, research on lift detection has not been deeply investigated and the number of related studies is limited (Naruse et al., 2003; Kawai et al., 2004). Kawai et al. proposed a myoelectric controller for a power assist device (Kawai et al., 2004). Artificial neural network (ANN) was used to process electromyographic (EMG) signals measured from three front and back thigh muscles. The controller could detect user's lift intentions and automatically output power assistance without control buttons. The limitation of this approach is that extra electrodes need to be placed on user's body, which could be not acceptable for workers in real scenarios (e.g., on the production line). Furthermore, EMG signals usually vary over time due to muscle fatigue, sweats, electrode displacement, skin conductivity changes, and other factors, which could influence the performance of lift detection. The optimal lift detection system for exoskeleton control should not only make accurate and timely detection of lift intentions, but also be simple and well integrated with exoskeleton devices, which is crucial for practical application. However, although there are some works using exoskeleton signals to detect other locomotion tasks (e.g., walking, stair ascent and descent, sitting down, and standing up) (Parri et al., 2017), to the best of our knowledge, there are no existing studies about the development of lift detection algorithms which use the signals from the exoskeleton's onboard sensors.

In this paper, we proposed a simple rule-based lift detection strategy based on sensors embedded in an active pelvis orthosis (APO). The algorithm is designed to detect lift movement at the very beginning of the lifting procedure, namely as soon as the user starts performing the lift movement. The set of sensors included two on-board encoders to measure hip joint angles of both sides and an inertial measurement unit (IMU) to measure kinematic signals of the trunk. To validate the algorithm for exoskeleton lift detection, seven healthy subjects were recruited. Parameters of the lift detection model were determined by data collected in two training sessions. During online test, the trained algorithm was used to detect the lift movement and control the exoskeleton to provide assistance. To evaluate whether the algorithm could be generalized, we also performed offline analysis of lift detection with subject-independent detection model. Timeliness of lift detection algorithm is important for real-time control of assistive exoskeletons, because it determines whether users can receive power assistance in time and further impact the performance of movement assistance. Therefore, apart from inquiring subject's subjective feedback on the timeliness of assistance supply, we also made quantitative evaluation of time delay of the detection. Finally, we assessed the effectiveness of the developed lift detection algorithm in conjunction with the delivery of assistive torque by measuring the EMG signals of three back muscles.

## Materials and methods

### Experimental setup

The experimental setup comprises: (i) the APO, (ii) an IMU, and (iii) a commercial EMG recording system.

**Figure 1:**
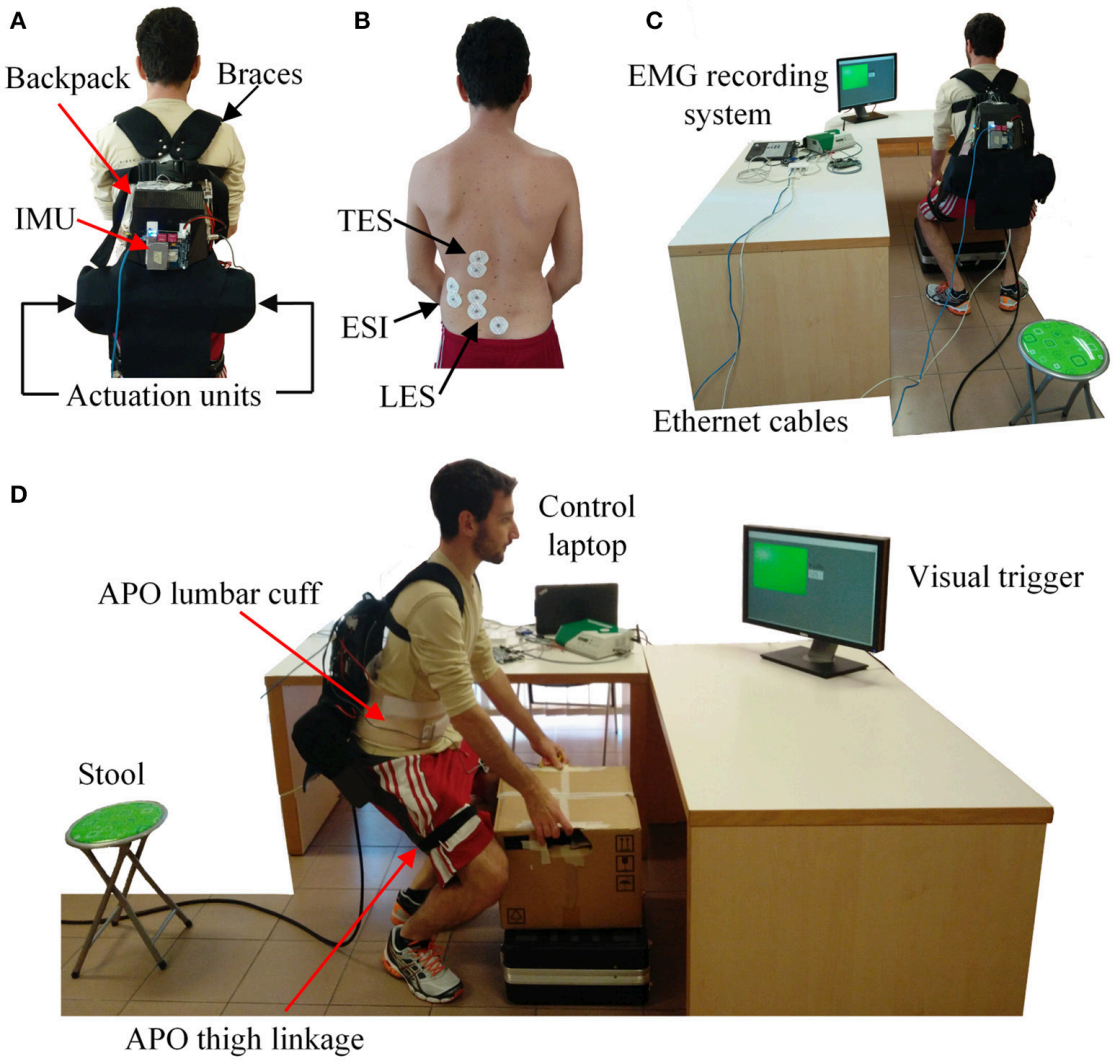
Experimental setup of this study. **(A)** A subject wearing the APO endorsed with an IMU on the back. **(B)** Electrodes placement for targeting the Lumbar Erector Spinae (LES), the Erector Spinae Iliocostalis (ESI) and the Thoracic Erector Spinae (TES). **(C)** Back view of the experimental setup. **(D)** Lateral view of the experimental setup. The subject provided written informed consent for the publication of this image.

The APO is a light-weight lower-limb exoskeleton for the assistance of the hip flexion/extension movement (Figure 1A). The exoskeleton used in this study is an improved version of the one presented in Giovacchini et al. (2015) and has been developed by the Wearable Robotics Laboratory of The BioRobotics Institute (Scuola Superiore Sant'Anna, Pisa, Italy). It is composed of (i) a frame structure connected to the user's trunk by means of straps and braces, and (ii) two rotating linkages connected with the user's thighs. The two links are actuated by means of two series elastic actuator units, which are able to provide up to ±22 N·m of peak torque. In this study, we took advantage of the torque delivered by the actuators to extend the hips to provide also an extension torque to the trunk to assist the lifting movement.

The APO controller architecture is hierarchical and developed on two levels. The high-level layer runs at 100 Hz and hosts the assistive strategy, i.e., the way to set the desired torque profile. The APO can be controlled in two different modalities, namely in transparent mode (TM, i.e., the exoskeleton is providing no assistive torque, but is fully transparent to user's movements), and in assistive mode (AM, i.e., the exoskeleton is delivering the torque to assist the movement). The low-level layer is a field programmable gate array (FPGA) running at 1 kHz; it is in charge of controlling the torque delivered by the actuators. The assistive strategy is designed to deliver an assistive torque based on hip joint kinematics; the delivery of the torque starts accordingly to the detection of the lifting movement by means of the lift detection algorithm. The assistive torque is computed according to (1):

(1) $$\{\begin{array}{c} \theta_{mean}=\frac{\theta_{R}+\theta_{L}}{2}\\ \tau_{d}=-(A\cdot \theta_{mean}+B\cdot {\dot{\theta }}_{mean}+C) \end{array}$$

where θ_*R*_ and θ_*L*_ are respectively the right and the left hip joint angles, θ_*mean*_ is the averaged joint angle, ${\dot{\theta }}_{mean}$ is the first derivative of the averaged joint angle, τ_*d*_ is the resulting desired torque. *A, B, C* are model constants, which are adjusted according to subject's preference on power assistance. The sign of the torque is the opposite of the joint angle. An example of the assistance torque supplied by the APO is shown in **Figure 3C**.

An IMU is placed on the backpack of the exoskeleton (Figure 1A). A sensory fusion algorithm is used to calculate Euler angles using raw IMU signals. The roll angle describes the movement of the trunk in the sagittal plane and it is used in the algorithm of lift detection. Note that IMU signals are only used for lift detection in this research. Sampling rate of all sensors is 100 Hz.

Electromyography (EMG) was recorded by means of a TeleMyo 2400R EMG recording system (Noraxon Inc., AZ, USA). Pre-gelled bipolar Ag/AgCl surface electrodes (Pirrone & Co., Milan, Italy) were used to collect EMG signals. Recorded EMG signals were sampled at 1.5 kHz and low-pass filtered at 500 Hz by the EMG recording system.

The whole system communicates through a UDP link. The three devices (i.e., the APO, the IMU, and the EMG recorder) were connected by Ethernet cables through a switcher to the main control laptop. In this way, it was possible to save all data synchronously.

The setup also comprises a 5-kg box, a 40-cm stool, a 90-cm table, a 20-cm raised stand from the ground, and a screen placed on the table in front of the subjects displaying an intermittent LED pacing the motion (Figures 1C,D).

### Experimental protocol

Seven healthy male subjects participated in this study (27.9 ± 2.3 years old, 178.1 ± 8.1 cm, 70 ± 6.4 kg). Experiments were carried out at the premises of The BioRobotics Institute of Scuola Superiore Sant'Anna (Pontedera, Italy). The research was approved by the local ethical committee and was conducted in accordance with the principles stated in the Declaration of Helsinki.

Before starting the experiment, the subjects were prepared for EMG signals measurements (Figure 1B). Surface electrodes were placed on the left side of the back over three muscles: Lumbar Erector Spinae (LES), Thoracic Erector Spinae (TES) and Erector Spinae Iliocostalis (ESI). Electrodes were placed following the SENIAM guidelines (Hermens et al., 2000). Then, two different maximum voluntary contractions (MVCs) were recorded against a resistance for 5 s and 1 min apart (Frost et al., 2009). The maximum value of the recordings was used as a reference to normalize the EMG signals (Frost et al., 2009).

The experiment consisted of three sessions. In the first two sessions, the APO was used in TM, so that subjects could move freely without perceiving any resistance to their movements. Data were collected for the training of lift detection model and offline analysis. The third session was performed for online evaluation: the APO was used in AM and torque assistance delivery was automatically triggered by the lift detection module. In this experiment, subjects were asked to lift a 5-kg box, and a visual interface was designed to pace the lifting and lowering actions.

#### Session 1

Subjects were asked to perform the following tasks and repeat them 10 times in each experimental trial. Once the visual interface displayed a visual cue, subjects were instructed to lower the body to reach the load on the stand, lift it up and put it on the table, return to stand-up posture and wait for the next visual cue; after a new visual cue was displayed, they were then instructed to reach the load on the table, lower it down and placing it on the stand, and finally return back to stand-up posture. While the pace of lifting/lowering was determined by the visual cue, the velocity of the lifting/lowering movement was at the user's self-selected speed. A total of five lifting conditions with different lifting techniques were performed (Table 1), meaning 5 experiment trials, with only 1 lifting condition tested per trial.

**Table 1:** Lifting conditions in session 1 and 2.

**Index of different lifting conditions**	**Initial position**			**Lifting technique**		
	**Front left**	**Front**	**Front right**	**Stoop**	**Squat**	**Freestyle**
1		√			√	
2		√		√		
3		√				√
4	√					√
5			√			√

*Stoop lifting is the one in which the knee joints are almost fully extended and the hip joints and vertebral column are flexed to reach the load, while squat lifting is the one in which the knee joints are fully flexed and the trunk is held as vertical as possible (Burgess-Limerick, 2003). Freestyle lifting is subject's preferred technique when performing the lifting task*.

#### Session 2

**Figure 2:**
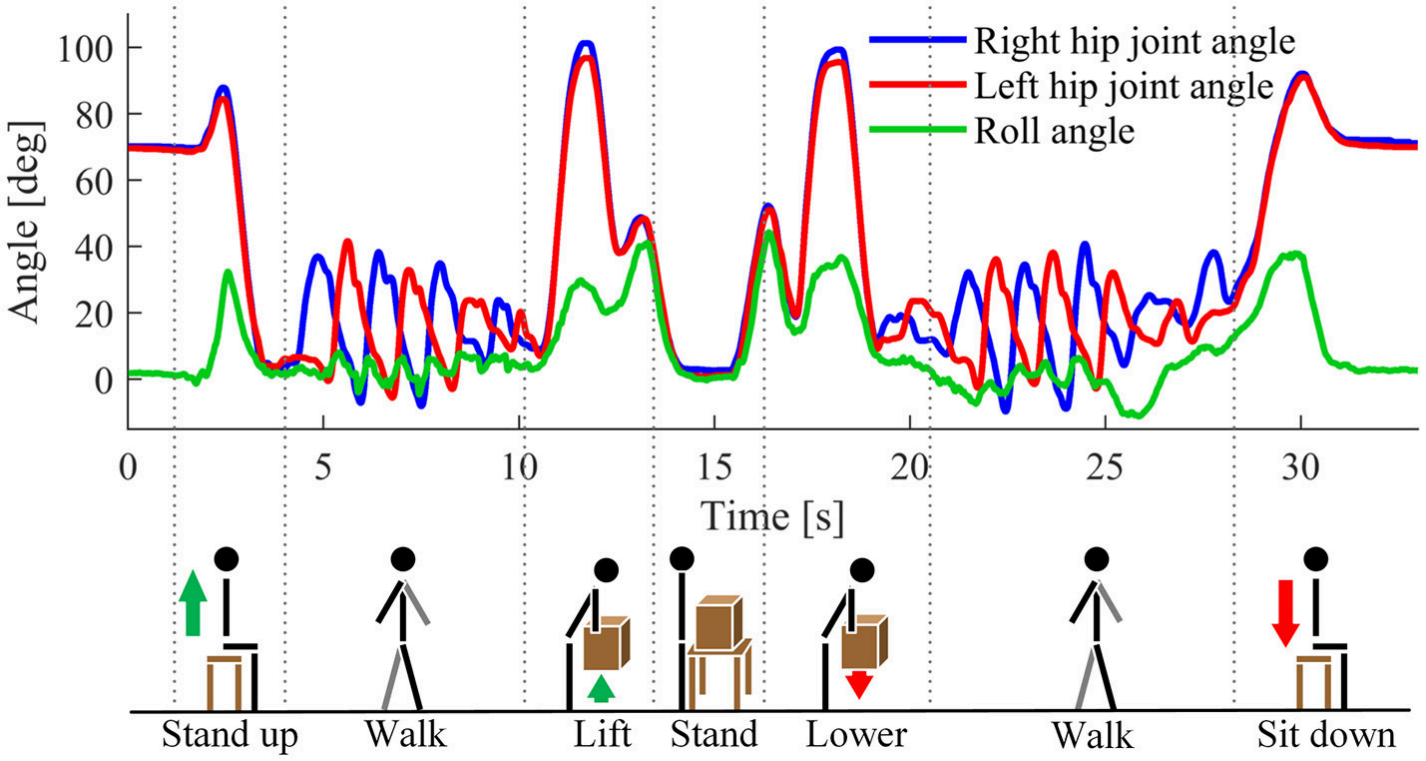
Signal measurement for one representative trial of session 2 along with a graphical representation of the performed task. Blue and red curves denote hip joint angle of the right and left side, respectively. Green curve denotes the roll angle measured by the IMU on the backpack.

Subjects were asked to perform the following movement sequence in each experiment trial: starting from a sitting position, standing up, walking toward the load, performing lifting and lowering tasks mentioned in session 1 (only once), walking back to the seat and sitting down (Figure 2). A total of 20 experimental trials were performed and each lifting condition was tested four times.

#### Session 3

The sequence of movements was the same as in session 2, with the main difference that subjects performed only “freestyle” lifting tasks with the box placed in front of them (i.e., condition 3 in Table 1). Instead of performing a single lift, subjects had to perform three repetitive lifts in each trial. In addition, in order to verify the robustness of the algorithm against different lifting speeds, subjects were asked to perform lift tasks at normal, slow and fast speeds. In particular, subjects were asked to choose their self-selected lifting speed, which was labeled as “normal”; then, “slow,” and “fast” speeds were obtained by asking subjects to perform lifting slower and faster than in normal speed, respectively. A total of 30 experiment trials were performed, with 10 trials performed for each lifting speed.

### Lift detection algorithm

**Figure 3:**
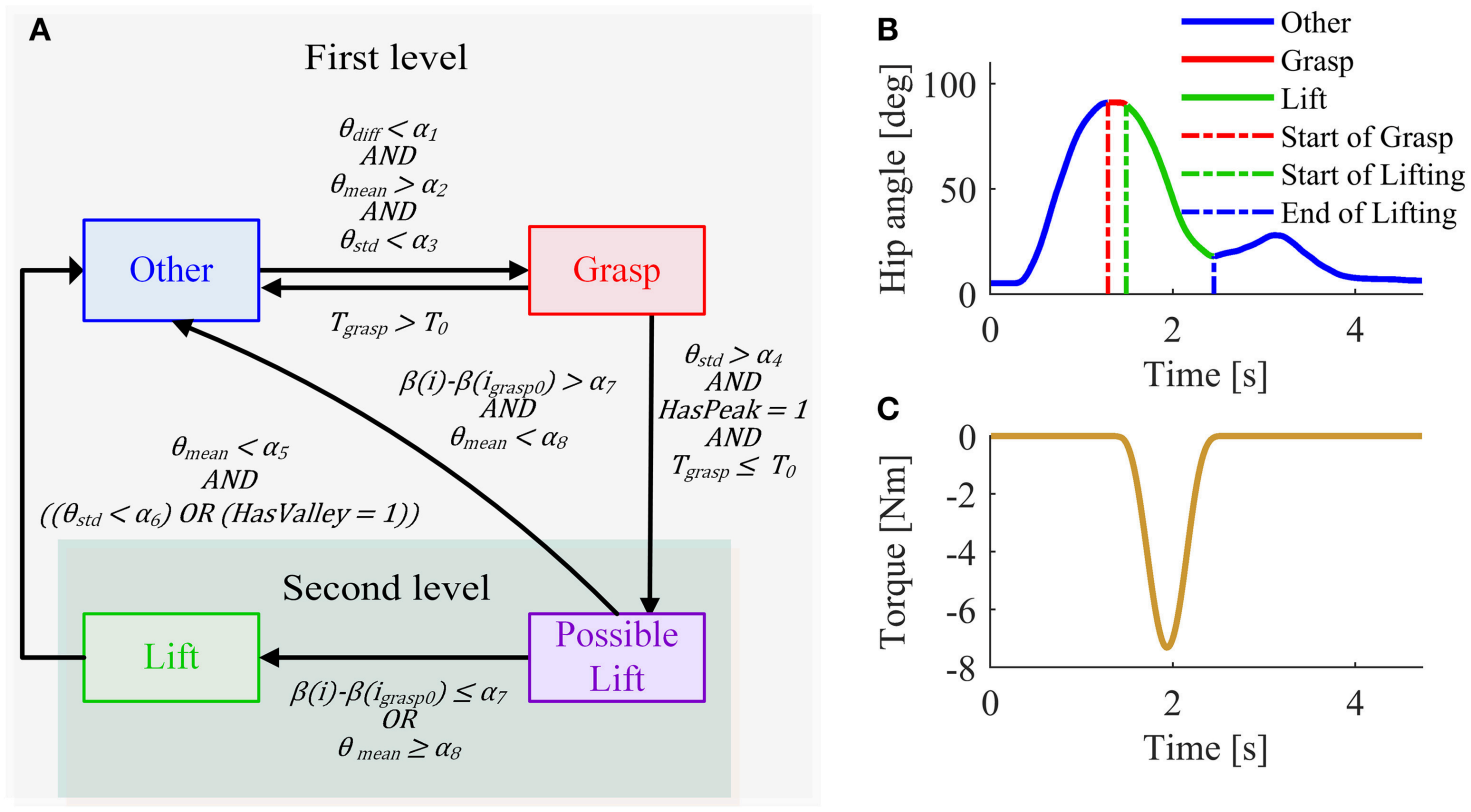
**(A)** Block diagram of lift detection algorithm. The light region denotes the first level of the algorithm, while the dark region denotes the second level. The second level of the algorithm is only performed when lifting is detected in the first level. θ_*diff*_ is defined as |θ_*L*_ − θ_*R*_|, where θ_*L*_ and θ_*R*_ denote hip joint angle of the left and right side, respectively. θ_*mean*_ is defined as (θ_*L*_ + θ_*R*_)/2 and θ_*std*_ is the standard deviation of (θ_*L*_ + θ_*R*_) over the last 100 ms. *T*_*grasp*_ is the duration of current grasping. β = θ_*mean*_ − φ, where φ is roll value of the IMU on the backpack of the exoskeleton. β could be considered as a rough estimation of thigh angle in the sagittal plane. *i* denotes the current sample and *i*_*grasp*0_ denotes the sample at the initial moment of grasping in current lifting. HasPeak equals to 1 if a peak of (θ_*L*_ + θ_*R*_) has occurred within the grasping phase, otherwise it equals to 0. HasValley equals to 1 if a valley of (θ_*L*_ + θ_*R*_) is detected within the lifting phase, otherwise it equals to 0. α_1_ to α_8_, and *T*_0_ are predefined thresholds. Note that α_1_, α_7_ and α_8_ are adjusted according to the training data, while the other thresholds are unchanged for different subjects. **(B)** Phase definition in the first level of lift detection algorithm. **(C)** Assistance torque supplied by the APO when a lift is performed.

A block diagram depicting the lift detection algorithm is shown in Figure 3A. It is a two-level rule-based algorithm. In the first level, the algorithm aims at detecting the possible moment of lifting using hip joint angles alone. In the second level, the algorithm aims at re-checking whether the detected lifting is a real one or represents other activities (e.g., sitting or walking) that could be mistakenly detected as lifting. Hip joint angles of both sides and Euler angles measured by the IMU on the trunk are used in the second level of the algorithm. Details of these two levels are described as follows.

#### First-level strategy

The first-level strategy was designed by assuming that before lifting happens, there is usually a grasping action. Therefore, the first level of the algorithm divides the lifting tasks into three main phases: *Grasp, Lift*, and *Other* (Figure 3B). Since these three phases happen sequentially, several rules are designed to detect transitions between them (Figure 3A).

##### Other–Grasp transition

In the *Grasp* phase, the hip joint reaches peak flexion angle and the user holds the grasping posture for a while (at least several hundred milliseconds), resulting in small hip joint angle variance. Therefore, the detection of the transition from *Other* phase to *Grasp* phase follows two main rules: (1) the average hip joint angle of both sides θ_*mean*_ is larger than a flexion angle α_2_, and (2) the standard deviation of hip joint angle θ_*std*_ is smaller than a predefined threshold α_3_. Note that flexion is defined positive and extension negative. In addition, since hip joint angles of both sides are usually very similar during symmetric lifting a third rule was used to detect the *Grasp* phase: (3) the absolute difference value of left hip joint angle and right hip joint angle θ_*diff*_ is smaller than a threshold value α_1_. This rule helps to avoid mistakenly detecting some asymmetric locomotion tasks (e.g., walking) as lifting.

##### Grasp–Lift transition

In the lifting phase, the hip joint starts to extent and move from a position corresponding to maximum flexion to the stand-up posture, resulting in a larger extension angular velocity than in grasping phase. Therefore, the detection of transition from *Grasp* phase to *Lift* phase follows two main rules: (1) the peak of hip joint angle has occurred, and (2) the standard deviation of average hip joint angle of both sides should be larger than a predefined threshold α_4_. Generally, the *Grasp* phase is very short in time. To reduce the impact of possible false detection of *Grasp* phase, the detection of *Lift* phase has also to satisfy a third rule: (3) the duration of current *Grasp* phase is shorter than a threshold *T*_0_; otherwise, current phase is interpreted as *Other*.

##### Lift–Other transition

Apart from *Grasp* phase and *Lift* phase, the rest is defined as *Other* phase. At the end of *Lift* phase, the hip joint angle reaches the maximum extension posture. From the observation of the hip joint angle profile, we noticed that the hip joint angle has much smaller variance than in *Lift* phase or that a valley value of hip joint angle occurs. Therefore, the detection of transition from *Lift* phase to *Grasp* phase has to satisfy the following rules: (1) the average hip joint angle of both sides θ_*mean*_ is smaller than a predefined threshold α_5_, and (2) the standard deviation of hip joint angle θ_*std*_ is smaller than a threshold α_6_ or the valley of hip joint angle has been detected.

#### Second-level strategy

In preliminary experiments, we found that subjects sometimes extend their trunks at a relatively fast speed immediately after sitting down. Because of the similarity between hip joint angles observed during lifting and trunk extension after sitting (Figure 2), using the information from only hip joint angles can cause erroneous lift detection. Therefore, the second level of the algorithm needs to be performed using the additional information provided by the IMU; additional detection rules were implemented. It is worth noting that the second level of the algorithm is only performed when a lift movement is detected in the first level: the “lift” detected in the first level is labeled as “possible,” and an intermediate phase is defined as *Possible Lift*. If the detection rules are satisfied in the second level, the “possible lift” is confirmed as “real lift,” namely the phase passes from *Possible Lift* to *Lift*; otherwise, the decision flow goes to Other phase.

**Figure 4:**
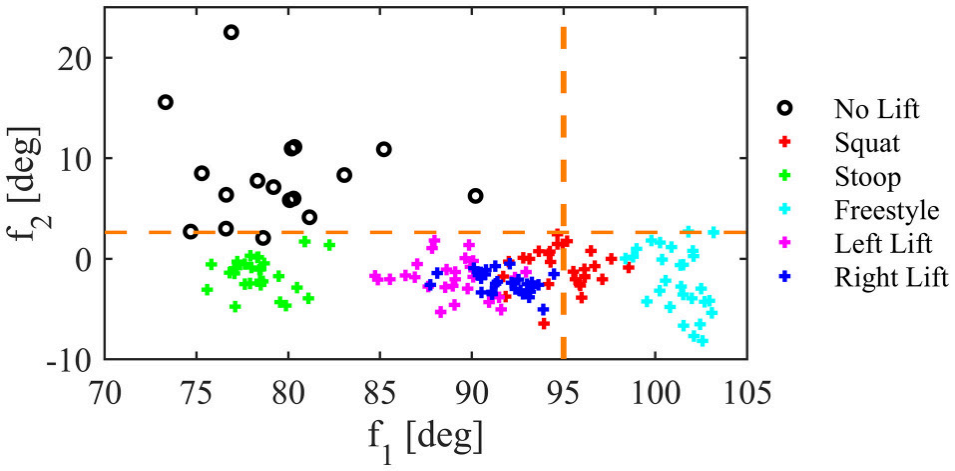
Features distribution for one representative subject for sitting (No lift) and lifting with different techniques. Orange dashed lines show thresholds identified for *f*_1_ and *f*_2_.

The second level of the algorithm exploited hip joint angles and IMU roll angle to estimate the thigh angle. Based on the thigh angle we used the following two features: *f*_1_ = θ_*mean*_ and *f*_2_ = β(*i*) − β(*i*_*grasp*0_), where θ_*mean*_ is the average hip angle of both sides and β is defined as θ_*mean*_ − φ (φ is roll value of the IMU on the backpack of the exoskeleton, *i* is the current iteration and *i*_*grasp*0_ is the iteration when *Other-Grasp* transitions occurred). Feature distribution of sitting and lifting with different techniques are shown in Figure 4 for one representative subject. To confirm the “possible lift” as “real lift,” the following two rules have to be met: (1) *f*_1_ ≥ α_8_, and (2) *f*_2_ ≤ α_7_, where α_7_ and α_8_ are two additional thresholds.

### Data analysis

#### Lift detection performance evaluation

To evaluate the reliability of the lift detection strategy, accuracy, precision and recall values were calculated. Their definitions are provided in the following Equations (2–4):

(2) $$\begin{array}{r} Accuracy=\frac{tp+tn}{tp+fp+tn+fn} \end{array}$$

(3) $$\begin{array}{r} Precision=\frac{tp}{tp+fp} \end{array}$$

(4) $$\begin{array}{r} Recall=\frac{tp}{tp+fn} \end{array}$$

where *tp* is the number of true-positive, which denotes lift correctly detected as lift; *fp* is the number of false-positive, which denotes non-lift activity mistakenly detected as lift; *tn* is the number of true-negative, which denotes non-lift activity not detected as lift; *fn* is the number of false-negative, which denotes lift not detected as lift. Note that whether lift and non-lift actions are correctly detected is manually determined by observing the raw signals.

Three different assessments of the lift detection performance were carried out for: (i) preliminary evaluation of the performance of subject-dependent training, (ii) online testing the algorithm after subject-dependent training and for (iii) offline evaluating subject-independent performances. In the first two analyses, the thresholds used by the rule-based algorithm were set for each subject, based on training data, whereas the algorithm was tested in training data and session 3 data respectively for (i) and (ii). In the third analysis, we set the same thresholds for all the subjects based on the observation of all training data from all subjects, and then tested on session 3 data. To compare the performance of subject-dependent and subject-independent lift detection, paired-samples *t*-test analysis was performed.

To quantitatively evaluate the timeliness of the lift detection strategy, time delay of lift detection was calculated for session 3 data. Since we did not have external sensors to determine the initial moment of lifting, we defined the time delay as the duration from the time instant of hip flexion angle peak to the one corresponding to lift being detected by the algorithm. One-way repeated measures ANOVA was performed to investigate the influence of lifting speed on time delay.

#### EMG analysis

The collected EMG signals were high-pass filtered (3rd-order Butterworth filter, 20 Hz cut-off frequency), rectified, and low-pass filtered (3rd-order Butterworth filter, 2 Hz cut-off frequency) to obtain the enveloped signal. Finally, the EMG signals were normalized by the maximum of the MVC. The integral of the EMG (iEMG) was computed according to Equation 5 over the time window of correct lifting detection:

(5) $$\begin{array}{r} iEMG=\Delta t\cdot \sum_{i=1}^{N}X_{i} \end{array}$$

where *X*_*i*_ is the *i*_*th*_ sample of the signal, *N* is the number of samples in the epoch and Δ*t* is the integration step.

iEMG was computed to assess whether the assistive strategy (namely the combination of lift detection and assistive torque) could reduce the muscular effort requested to back extensor muscles during the lifting action. iEMGs were averaged across subjects and paired-samples Wilcoxon signed-rank test was used to check for differences among transparent and assistive conditions.

Data analysis and statistics were performed in Matlab 2017 (The Mathworks, Natick, USA). All statistical analyses were considered significant for *p* < 0.05.

## Results

### Training performance of subject-dependent lift detection algorithm

To have a basic understanding of whether the lift detection model was well trained, it was offline tested on the training data. Averaged across all the subjects, accuracy was 99.38 ± 0.43%, precision was 99.5 ± 0.3%, and recall was 99.8 ± 0.2%. Most of the errors were mistaken detection of sitting down as lifting.

### Online evaluation of subject-dependent lift detection algorithm performance

**Figure 5:**
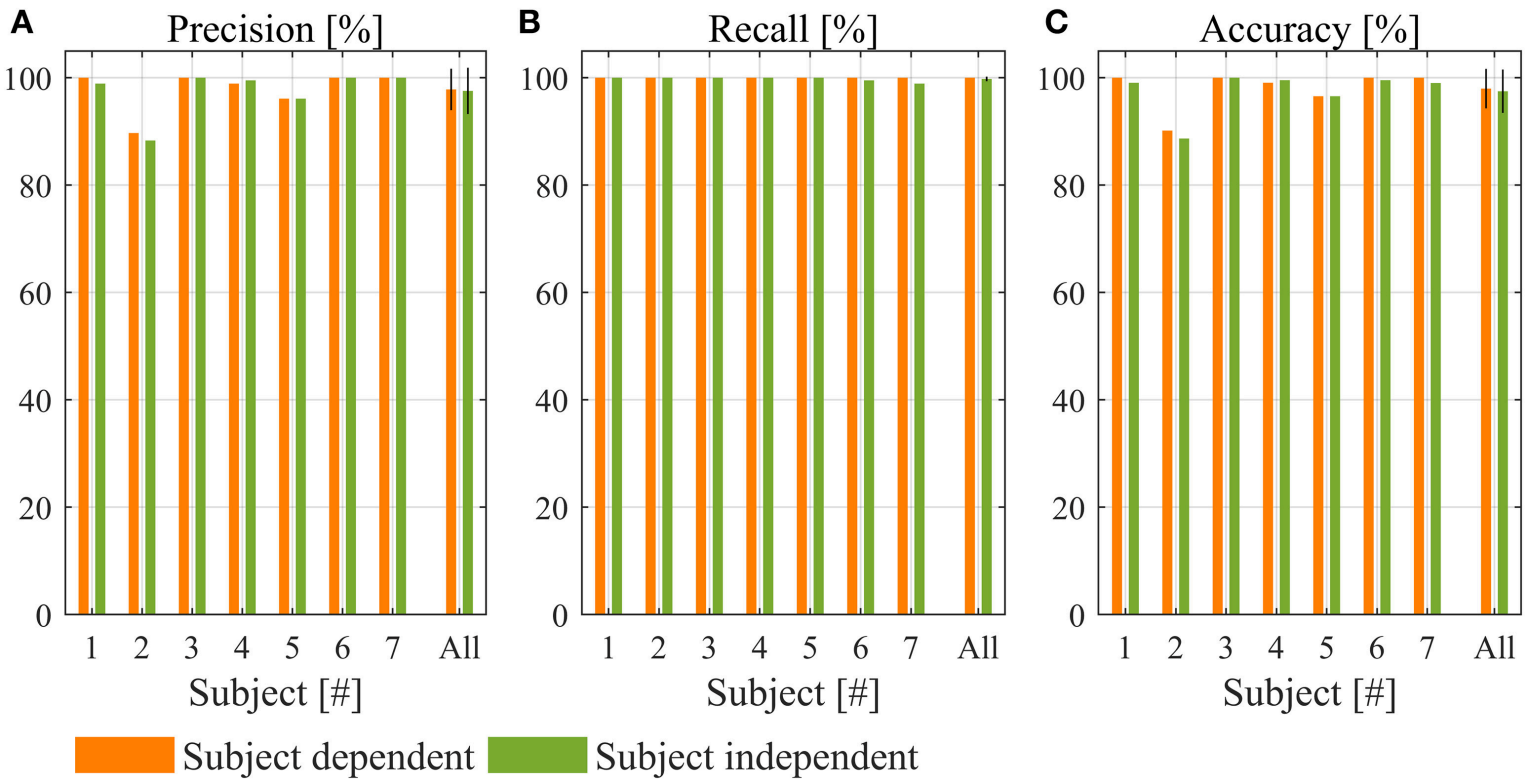
Performance of lift detection. **(A)** Precision, **(B)** recall, and **(C)** accuracy for subject-dependent and subject-independent algorithm.

**Figure 6:**
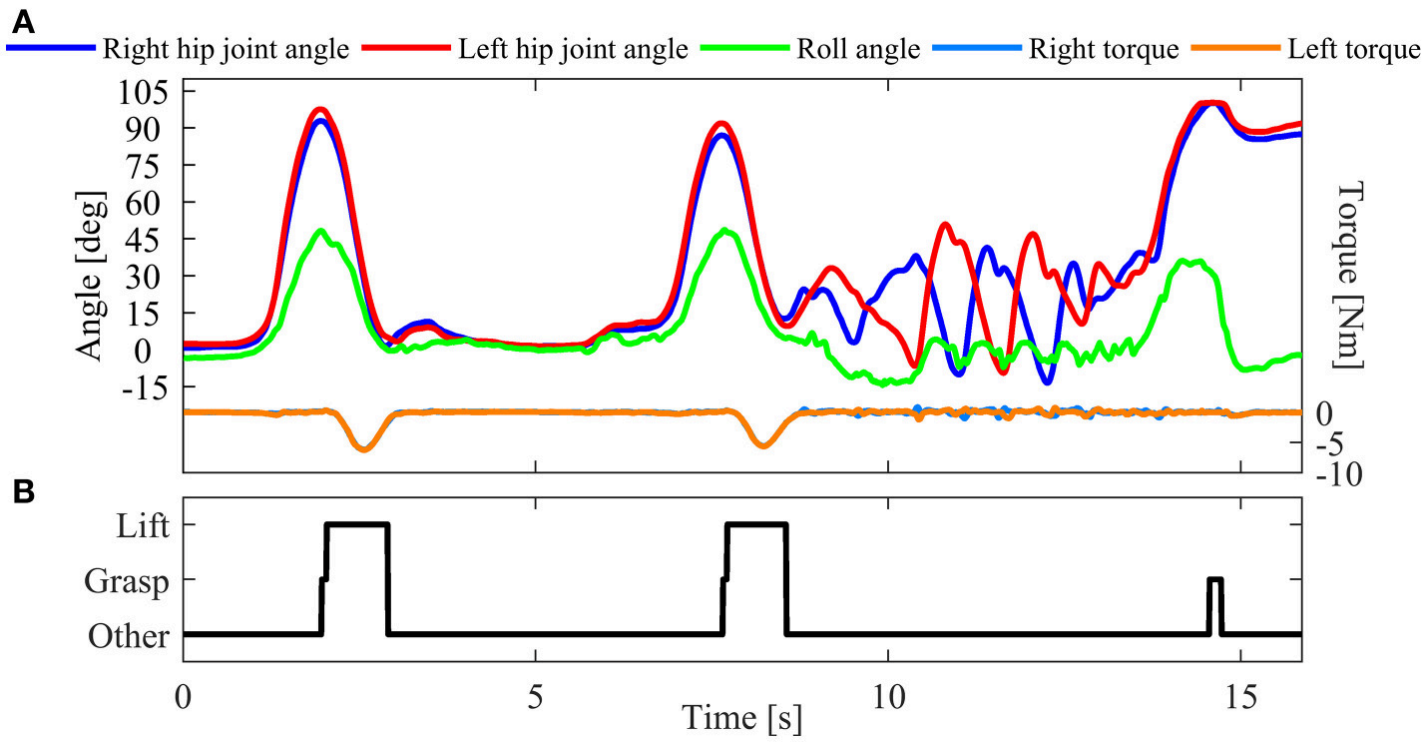
An example of online verification of the lift detection algorithm (session 3 of subject 1). **(A)** Hip joint angles, IMU roll angle and assistance torque recorded during online test. **(B)** Result of online lift detection.

Averaged across all subjects, accuracy was 97.97 ± 1.39%, precision was 97.8 ± 1.5%, and recall was 100% (Figure 5). No miss detection of lifting was observed for all subjects. An example of online test is shown in Figure 6.

### Offline evaluation of subject-independent lift detection algorithm performance

Averaged over all the subjects, accuracy was 97.48 ± 1.53%, precision was 97.5 ± 1.6%, and recall was 99.8 ± 0.2% (Figure 5). Compared to the performance of subject-dependent lift detection algorithm, accuracy, precision and recall reduced by 0.49, 0.3, and 0.2%, respectively. However, none of them was statistically significant (*p* = 0.110 for accuracy, *p* = 0.350 for precision, and *p* = 0.207 for recall).

### Time delay of lift detection

**Figure 7:**
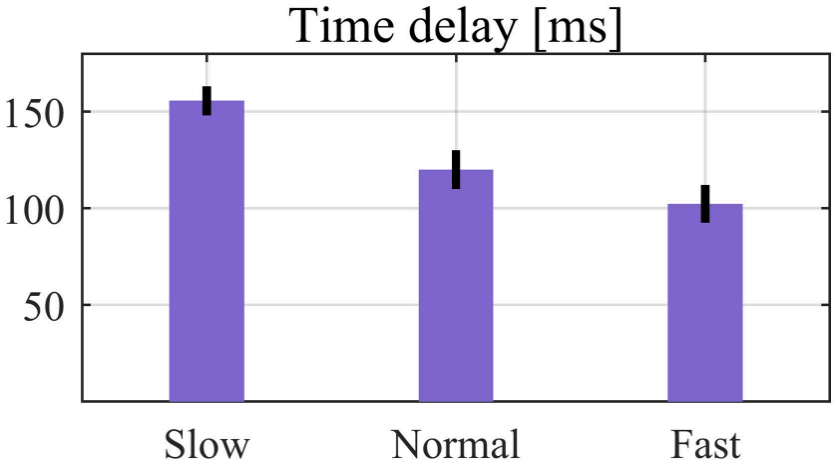
Average time delay of lift detection over seven subjects. Error bars denote SEMs across subjects.

For all subjects there was no significant delay in torque delivering when lifting tasks were performed. Note that only correct lift detections were considered for time delay calculation. The values of the time delay changed along with the lifting speed (Figure 7): 156 ± 7 ms, 120 ± 10 ms, and 102 ± 10 ms for slow, normal and fast speed, respectively. The impact of lifting speed on time delay was statistically significant (*p* < 0.05 for all pair-wise comparisons). The average values of slow, normal, and fast lifting speed across subjects were 74.8 ± 7.3 deg/s, 109.1 ± 5.2 deg/s, and 130.4 ± 4.6 deg/s, respectively. The lifting speed is defined as the average hip joint angular speed from the moment of hip angle peak to the end of the lifting procedure (detected by the algorithm).

### EMG results

**Figure 8:**
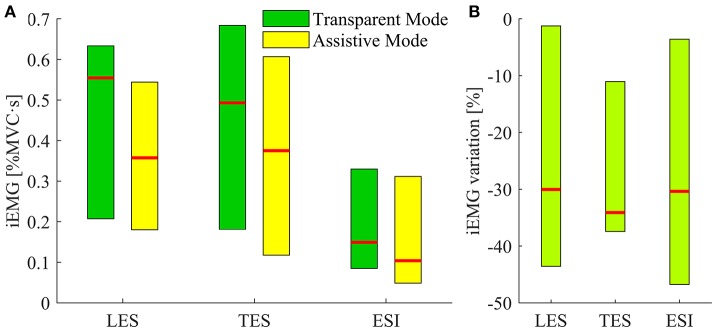
iEMG results. **(A)** Bars depicting the iEMG median values distribution for the subjects' three muscles while wearing the APO operating in transparent mode (i.e., session 1 and 2, green), and assistive mode (i.e., session 3, yellow). Red lines correspond to the median values, upper and lower limit of the bars are respectively the maximum and the minimum values of median iEMG. Data for subject 1 and 2 are missing because of an operating failure of the EMG recording device during the experimental sessions. **(B)** Bars pointing at the percentage variations of the iEMG values in assistive mode with respect to the median values of transparent mode for the three muscles under investigation. Upper and lower limits of the bar are respectively the minimum and maximum variation achieved among subjects.

Figure 8A shows the iEMG computed during the trunk extension movement for the three targeted muscles. Comparison between TM and AM conditions is reported. All muscles showed significant reductions of the iEMG median value during AM with respect to TM (*p* < 0.05): −30% for LES, −34.1% for TES, and −30.4% for ESI (Figure 8B).

## Discussion

Intention detection is fundamental for the control of assistive exoskeletons, determining whether a user can receive appropriate power assistance from the exoskeleton at the optimal moment. In this study, a lift detection strategy was developed for exoskeleton control aiming at lift assistance. The proposed algorithm has the following advantages that could make it promising for practical application in factories and other working scenarios (e.g., personal care workers, nurses).

First, the algorithm only employed sensory information from signals collected with exoskeleton embedded sensors: this property of our algorithm could make the overall system more compact and convenient to use during working, thus fostering the use of exoskeletons in real industrial environments. As we already mentioned, the number of studies exploring lift detection techniques and algorithms is limited. To the best of our knowledge, in all these works off-robot signals were employed to trigger exoskeleton assistance delivery. For example, one of the most used strategy relies on EMG signals (Naruse et al., 2003; Kawai et al., 2004). EMG signals have the potential to be directly related to the human movement, thus different control strategies can be implemented. Nevertheless, the use of EMGs is not trivial in industrial scenarios, due to possible acceptability issues from the workers. The need of wearing electrodes and corruption of the signal over time due to sweat are only some of the possible drawbacks (Sensinger et al., 2009). Furthermore, these studies did not report systematic validation of lift detection algorithms and results were only at a preliminary stage. Compared to the algorithms based on EMG signals, our algorithm does not need to use additional sensors that cannot be embedded with the exoskeleton, which makes it more promising for practical use. In addition, the proposed algorithm is a simple rule-based one, which has much lower computation burden compared to more complex recognition methods, such as deep-learning, and it could be easily implemented in the real-time controller of the robot without affecting the computational cost. Therefore, the timeliness of lift detection can be guaranteed.

Second, our algorithm is able to reliably detect lift intentions with an accuracy higher than 97%. Furthermore, the algorithm is robust to different lifting techniques and lifting speeds, which makes it suitable to be used in different lifting tasks. For different lifting techniques, kinematic signals change a lot. For example, squat lifting has larger range of movement (RoM) in the knee but smaller RoM in the trunk, whereas stoop lifting shows the opposite behavior (de Looze et al., 1993). Moreover, inter-subject variability adds another degree of complexity. These kinematic differences introduce additional challenges to implement reliable lift detection systems. To evaluate the robustness of the lift detection strategy to different lifting tasks, subjects were asked to lift the load under different lifting conditions (e.g., different lifting techniques and lifting speeds). Training performance (lift detection test with the training data) showed that the algorithm was able to overcome the impact of different lifting techniques and achieved an average detection accuracy of 99.38%. For simplicity sake, we tested the current lifting assistance strategy only with freestyle lifting. However, according to the training performance, we believe the lift detection algorithm can also work well together with assistive strategies for other lifting techniques.

Third, time delay of lift detection is small and will not introduce any significant delay of the assistance supplied by the exoskeleton. To provide timely assistance to users the lift detection algorithm has to detect the onset of the movement as soon as it occurs, otherwise the exoskeleton action could be not properly synchronized with the movement to be assisted. Quantitative results showed that time delay was related with the lifting speed. However, even for the slow-speed lifting, no obvious time delay of assistance supply was reported. In fact, although a small time delay was observed, subjects could still receive proper assistance from the exoskeleton as demonstrated by reductions in EMG activities of back muscles. In addition, despite the existence of such small detection delay, being the exoskeleton controlled in TM in movements other than lifting, a comfortable human-robot interaction was always ensured.

Fourth, the algorithm has the potential to be subject-independent, which means it could work with new user without any training. According to our findings, we observed the subject height as the main factor influencing lifting kinematics for different subjects. In the existing subject pool, subject's height ranges from 165 to 186 cm, which covers the height of most male users, as the 5–95th percentiles ranges from about 163 to 188 cm (Fryar et al., 2012). Though many thresholds were used in the lift detection algorithm, we noticed that most of the thresholds did not need to be changed. In the subject-dependent lift detection algorithm, only α_1_, α_7_, and α_8_ were adjusted for different subjects, possibly due to anthropometric differences and sit-down behavior. Experimental results showed that the reduction of lift detection performance was limited when replacing the subject-dependent algorithm with the subject-independent one. Furthermore, the reduction was not statistically significant.

The EMG analysis revealed that the developed lift detection algorithm works effectively with the assistive strategy implemented in the APO. Indeed, the iEMG of muscles signal decreases while using the exoskeleton in assistive mode. The achieved reductions are comparable with those reported by a series of studies on the Muscle Suit exoskeleton (Kobayashi and Nozaki, 2007, 2008; Aida et al., 2009; Kobayashi et al., 2009; Muramatsu et al., 2011). However, it is fair to stress that the experimental protocol of this study did not include any experimental session without wearing the exoskeleton and, in addition, there was not any randomization of the experimental trials. This is due to the main focus on the development of the lift detection module, rather than assessing the effectiveness of an assistive strategy.

The main limitation of the lift detection strategy is that sometimes it still mistakenly detects sitting down as lifting for some subjects (e.g., subject 2 and subject 5). Mistaken detections are probably caused by the following reasons. First, since subjects took off the exoskeleton after experiment sessions for training and wore it again before testing sessions, the position of the exoskeleton might change a little and therefore influence readings of hip joint encoders and the IMU on the trunk. As the algorithm is sensitive to these information, mistaken detection could be caused by the wearing problem. Second, threshold values were determined by the data measured in the training sessions. If subjects changed their ways of sitting down a lot, mistaken detections could also happen, which could cause slight discomfort to users. However, in real working scenario, the chance to sit down with an exoskeleton is expected to be much lower than that in our experiment. In addition, for the main purpose of this work, we considered acceptable occasional misclassification of sitting down as lifting, since the main goal of this algorithm was to effectively recognize lifting tasks when they actually occurred.

Future works will be focused on improving the robustness and generality of the lift detection strategy on a larger number of subjects, especially for avoiding misdetection of sitting as lifting. In particular, possible new features will be introduced and used to validate the algorithm also for discriminating between different lifting techniques. In addition, we will focus more on assessing the real effectiveness of the developed assistive strategy in reducing the muscular effort of the posterior chain muscles.

## Conclusion

In this research, we proposed a simple rule-based lift detection strategy. The algorithm only used hip joint angles of both sides and trunk angle in the sagittal plane, which could be measured by exoskeleton embedded sensors. The algorithm was able to achieve reliable performance of lift detection and was robust to different lifting techniques and lifting speeds. In addition, time delay of lift detection was very small, which did not introduce noticeable discomfort when power assistance was provided to subjects. We also evaluated the generality of the algorithm applying on different subjects. The result of subject-dependent lift detection did not change significantly. Furthermore, by combining the developed lift detection algorithm with a simple assistive strategy, the EMG analysis revealed a general reduction of the back muscles activity, proving that the assistance provided by the exoskeleton is beneficial for the user in load lifting tasks. These results validated the promise of applying the lift detection strategy for the control of exoskeletons aiming at lift assistance.

## Ethics statement

This study was carried out in accordance with the recommendations of Scuola Superiore Sant'Anna ethics committee with written informed consent from all subjects. All subjects gave written informed consent in accordance with the Declaration of Helsinki. The protocol was approved by the Scuola Superiore Sant'Anna ethics committee.

## Author contributions

BC carried out the experimental activities and data analysis, participated in the design of the study and drafted the manuscript. LG and FL carried out the experimental activities, participated in the design of the study and drafted the manuscript. SC and NV conceived the study, participated in the design and coordination of the study. All authors approved the submitted version of the manuscript.

### Conflict of interest statement

SC and NV have commercial interests in IUVO S.r.l., a spin-off company of Scuola Superiore Sant'Anna. Currently, part of the IP protecting the APO technology described in the paper has been licensed to IUVO S.r.l. for commercial exploitation. The authors confirm that this did not affect the analysis of the results. The other authors declare that the research was conducted in the absence of any commercial or financial relationships that could be construed as a potential conflict of interest.
